# Exploring the role of space-defining objects in constructing and maintaining imagined scenes

**DOI:** 10.1016/j.bandc.2013.02.013

**Published:** 2013-06

**Authors:** Sinéad L. Mullally, Eleanor A. Maguire

**Affiliations:** Wellcome Trust Centre for Neuroimaging, Institute of Neurology, University College London, 12 Queen Square, London WC1N 3BG, UK

**Keywords:** Scenes, Objects, Space-defining, Parahippocampal cortex, Scene construction, Memory

## Abstract

It has recently been observed that certain objects, when viewed or imagined in isolation, evoke a strong sense of three-dimensional local space surrounding them (space-defining (SD) objects), while others do not (space-ambiguous (SA) objects), and this is associated with engagement of the parahippocampal cortex (PHC). But activation of the PHC is classically associated with scene stimuli. The comparable neural response within PHC to both full scenes and single SD objects, led us to hypothesise that SD objects might play a more critical role in the construction and maintenance of scene representations than SA objects. To test this we used scene construction and deconstruction paradigms, where participants gradually built and maintained scenes using SD, SA and background (wall, floors) items. By examining the order in which each item was added (and later removed) to (and from) a scene, we could estimate the significance of each item type. In two different experiments, participants chose SD over SA objects and background items as the first and most critical item in their constructed scenes and, more generally, selected SD objects earlier than SA objects across the scene construction process. When deconstructing scenes, participants retained significantly more SD objects than SA objects, and the last remaining object across all scenes was highly likely to be an SD object. SD objects therefore enjoy a privileged role in scene construction and maintenance, and appear to be an essential building block of scenes.

## Highlights

• Some objects evoke a sense of surrounding space (space-defining; SD).
• We explored their role in constructing and maintaining imagined scenes.
• SD objects enjoyed a privileged role in scene construction and maintenance.
• SD objects appear to be an essential building block of scenes.

## 1 Introduction

For many years, vision, cognitive, and neuro-scientists have studied the nature, perception and memory of scenes (Bar, 2004; Biederman, 1972; Biederman, Mezzanotte, & Rabinowitz, 1982; Bisiach & Luzzatti, 1978; Enns & Rensink, 1990; Epstein & Kanwisher, 1998; Henderson & Hollingworth, 1999; Intraub & Richardson, 1989; Mandler & Parker, 1976; Oliva & Schyns, 1997; Potter & Faulconer, 1975). The identification of a region in posterior parahippocampal cortex (PHC), which appears to be preferentially responsive to topographic information (Aguirre, Detre, Alsop, & D’Esposito, 1996) and scene stimuli (Epstein, Harris, Stanley, & Kanwisher, 1999; Epstein & Kanwisher, 1998), has focused much attention on the neural mechanisms underpinning scene processing. However, what specific scene attributes are represented within the PHC has been widely debated (Ranganath & Ritchey, 2012), and numerous hypotheses, which focus on different aspects of scenes, have been proposed. These include their spatial layout or global structure (Epstein, 2008; Epstein et al., 1999; Park, Brady, Greene, & Oliva, 2011; Walther, Chai, Caddigan, Beck, & Fei-Fei, 2011), contextual (Bar, 2004; Bar, Aminoff, & Schacter, 2008) or categorical (Naselaris, Prenger, Kay, Oliver, & Gallant, 2009; Walther, Caddigan, Fei-Fei, & Beck, 2009) information, scene novelty (Howard, Kumaran, Olafsdottir, & Spiers, 2011), or navigational relevance (Janzen & van Turennout, 2004). Moreover, the distinctive network of brain regions (which includes the PHC), activated when participants actively imagine complex, coherent scenes (Hassabis, Kumaran, & Maguire, 2007; Summerfield, Hassabis, & Maguire, 2010), recall episodic memories, plan for the future or engage in spatial navigation (Buckner & Carroll, 2007; Hassabis & Maguire, 2007; Schacter, Addis, & Buckner, 2008; Spreng, Mar, & Kim, 2009), suggests that understanding scene processing in regions such as PHC may be key to elucidating a range of cognitive functions (Hassabis & Maguire, 2007, 2009).

**Fig. 1:**
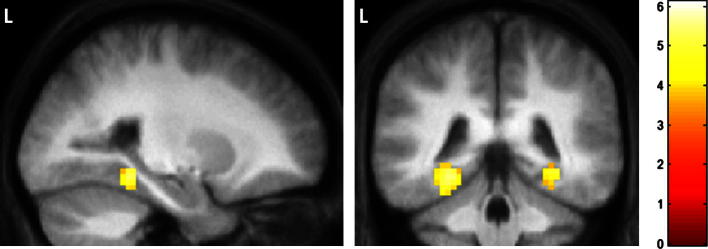
Brain areas engaged by imagining SD relative to SA objects. Activations at the level of the peak left PHC voxel are shown on sagittal (left panel) and coronal (right panel) images on the averaged structural MRI scan of the 21 subjects from the Mullally and Maguire (2011) study. The colour bar indicates the *z*-scores associated with each voxel. L = left side of the brain.

In a recent study Mullally and Maguire (2011) offered an alternative account of PHC function (see Kravitz, Peng, & Baker, 2011; and Doeller & Kaplan, 2011, for related discussions), proposing that the PHC is selectively engaged by representations that depict local three-dimensional space. Scenes, by their very nature, invariably encompass this. However, Mullally and Maguire (2011) reported that certain types of objects, when imagined or viewed in isolation, evoked a strong sense of three-dimensional local space surrounding them. Such objects were identified as ‘space-defining’ (SD) objects, whereas objects that did not evoke this impression were referred to as ‘space-ambiguous’ (SA) objects. Critically when the neural responses to these two object categories were compared, a robust signal in the posterior parahippocampal cortex was observed associated specifically with the SD objects (Fig. 1). Importantly, this response was not explained simply by object size alone (Konkle & Oliva, 2012) or contextual associations (Bar et al., 2008). The location of this PHC activation mirrored that typically observed when scene stimuli are compared to single objects (Epstein & Kanwisher, 1998; Epstein et al., 1999; O’Craven & Kanwisher, 2000). Thus, Mullally and Maguire (2011) argued that the three-dimensional space inherently present in scenes but also evident at a more local level in relation to SD objects, may represent the key attribute processed by the PHC (see also Zeidman, Mullally, Schwarzkopf, & Maguire, 2012).

**Fig. 2:**
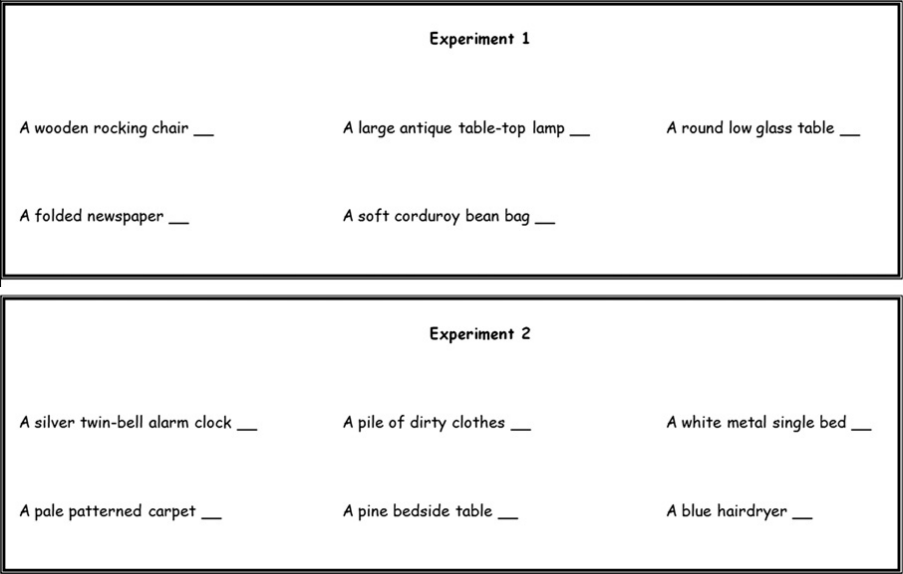
Example stimuli. Example stimulus for Experiment 1 comprised of SD and SA items, and for Experiment 2 comprised of SD, SA and background elements.

The question remains however, as to whether SD objects are behaviourally relevant to scenes in a way that SA objects are not. In order to examine the relationship between SD objects and scenes, we adapted a technique previously devised by Summerfield et al. (2010), where participants were required to construct indoor scenes in the mind’s eye, item-by-item. The items presented to participants were typical household objects and background elements (such as walls and floors). The incremental presentation of items ensured that the scene construction process was ‘slowed-down’ into distinct steps which could then be individually interrogated. Using this paradigm, we had participants construct scenes in the imagination, using a combination of SD and SA objects (Experiment 1), plus background items (Experiment 2). The order in which the items were presented was not predetermined. Instead, on each trial participants were presented with a written description of the items simultaneously, and constructed their scenes, item-by-item, while noting the order in which the items were added to their imagined constructions (Fig. 2). The participants’ overall goal was to achieve the impression of a real-world scene as early in the scene construction process as possible. Thus, by examining the order in which participants chose to add items into their constructions, the category of items considered to be the most influential in the construction of scenes was revealed. We hypothesised that SD objects would be selected earlier in the scene construction process than either SA objects (Experiments 1 and 2) or background items (Experiment 2). In addition, we asked participants to subsequently deconstruct their imagined scenes. This enabled us to examine which object category was most critical in the maintenance of scenes. Again, we predicted that as participants sought to preserve their scene constructs, SD objects would be retained more often than either SA objects or background items.

Despite the extensive research that has been performed on scenes over the last five decades this is, to our knowledge, the first study exploring how mentally generated scene representations are specifically constructed and maintained, and the SD/SA categorisation enabled us to elucidate the significance of three-dimensional local space in this process.

## 2 Materials and methods

### 2.1 Participants

Forty healthy volunteers participated in the study; 20 in Experiment 1 (11 females; mean age 25.5 years; StD 3.88) and 20 in Experiment 2 (15 females; mean age 25.7 years; StD 4.72). All participants had normal or corrected to normal vision, were fluent English speakers, and gave informed written consent in accordance with the local research ethics committee.

### 2.2 Stimuli

As part of the Mullally and Maguire (2011) study, descriptions of 399 everyday indoor items were rated for SD/SA across a series of behavioural experiments. The items used here were drawn from the stimuli that were rated as most strongly SD and SA in that study.

#### 2.2.1 Experiment 1

On each of 20 trials, participants were presented with written descriptions of five objects found in typical indoor household scenes (Fig. 2). Participants were initially required to imagine each of these five objects in isolation, and to later attempt to construct and subsequently deconstruct scene representations in the mind’s eye using these five objects (see the Sections 2.3 and 2.4 below). Thus, participants imagined a total of 20 scenes, 10 of which contained three SD objects and two SA objects (‘Type 1’) and ten of which contained two SD objects and three SA objects (‘Type 2’). By using equal number of both scene types we ensured that any preference for either SD or SA objects was not being driven by an over-representation of either object category in the given scenes. All objects were accompanied by one or two adjective descriptors (e.g. ‘a pine bedside table’). Participants were unaware of the SD/SA distinction.

#### 2.2.2 Experiment 2

The stimulus set from Experiment 1 was extended in Experiment 2 to include background items (Fig. 2). Ten scenes were now comprised of three SD objects and two SA objects (as before), plus one additional background item (e.g. ‘a dark stone floor’ or ‘a light yellow wall’; ‘Type 1’; whereby 5 trials included a wall element and 5 a floor element). Ten scenes involved two SD objects, three SA objects, and one background item (‘Type 2’; 5 trials included a wall element and 5 a floor element). An additional ten scenes were also included (‘Type 3’), which contained two SD objects, two SA objects, and two background items (1 wall element and 1 floor element on each trial). Again, all objects and backgrounds were accompanied by one or two adjective descriptors. As in Experiment 1, these new participants were unaware of the SD/SA distinction.

### 2.3 Scene construction

Participants had to incrementally construct scenes in the mind’s eye. In order to explain the term ‘scene’, participants were asked to imagine the waiting room in which they had just been sitting. They reflected upon the fact that what they imagined in their mind’s eye was not a collection of separate objects occurring in isolation and devoid of context, but instead a fully integrated scene that contained these items. They were informed that the goal of this research was to ascertain how the brain puts together all the individual items within a scene (such as objects, walls and floors) to enable us to imagine and attain these seamless impressions of scenes in our mind. They were therefore encouraged to focus their attention on the transition from imagining a collection of single items to imagining the items integrated together into a coherent scene. The task began with participants imagining each object, one at a time, in isolation, in as much detail as possible and against a blank background. This was followed by the scene construction process. Here, participants imagined a ‘clean slate’ (i.e. they imagined the first item without any background context) after which they gradually built up the scene, item-by-item, ideally ending with the impression of a fully integrated, realistic and coherent scene.

Each scene had to be constructed using the five (Experiment 1) or six (Experiment 2) individual items provided and no others. Participants were therefore explicitly informed that they were not permitted to imagine any additional items or structures in their constructed scenes. This was emphasised a number of times throughout the task instructions, as was the requirement to start each trial with a blank background. Furthermore, participants were informed that the ultimate goal of the task was to attain the impression that what they were imagining was a scene, or in other words, a space that they could plausibly encounter in the real world. Critically for the task at hand, participants were instructed that they should attempt to attain this goal as early in the construction process as possible. On each trial, a description of the items was presented in typed form, on one page of white A4 paper, divided into three columns (Fig. 2). In this way, all five (Experiment 1) or six (Experiment 2) items were visible simultaneously. The task was therefore to select the items which were most helpful to them in attaining this impression of a coherent scene (in contrast to isolated single objects). The first item (i.e. the item that the participant considered to be the most important in helping to construct the scene) was imagined in isolation and against a blank background and was marked ‘1st^’^ by participants on the scoring sheet. The second item (marked 2nd by participants) was therefore the item that participants considered to be the next most important. This second item was imagined and integrated with the first item that had already been imagined. In this way, the procedure was repeated until all five (Experiment 1) or six (Experiment 2) items had been integrated into the scene. Finally, it was emphasised prior to starting the task that participants should try to imagine the scenes being built up in front of them, as if they were standing in an open doorway, so that the distance between them and the items in the scene was constant and unchanging.

Trials where participants were unable to construct a scene using the items provided, or without adding in additional items/structures, were excluded (trials excluded: Experiment 1, 0%; Experiment 2, 1.67%). The items selected to be imagined first (and therefore considered to be the most influential and important in establishing the scene) were assigned the highest score (i.e. ‘5’ in Experiment 1 and ‘6’ in Experiment 2). The second selected item was assigned the second highest scores (i.e. ‘4’ in Experiment 1 and ‘5’ in Experiment 2) and so on, until the last selected item was assigned the lowest score (i.e. ‘1’ in both Experiments 1 and 2). The average scores associated with the SD and SA objects (and background items in Experiment 2) in each scene trial were then calculated, providing a ‘mean significance score’ for each item type in the scene construction process.

### 2.4 Scene deconstruction

Following the construction of all scenes (20 in Experiment 1 and 30 in Experiment 2), participants were presented with the same scenes (one at a time) and informed that this time they would be asked to both construct and then deconstruct each scene. The scenes were presented in exactly the same manner as before, with the exception that the presentation order of items differed from phase 1. Participants were told not to attempt to remember the order in which they had constructed each scene in the previous construction phase but to simply form a scene using only the described items. Construction order was not noted in this phase. The deconstruction process was then broken into two parts. Participants were first asked to identify and remove any items which they felt they could afford to take away from that scene without altering the scene’s vividness in their imagination, or compromising the sense that what they were visualising was a definite indoor scene. They indicated their selection by placing an ‘*x*’ beside the items they wished to remove from the scene, leaving only the core scene items, the combination of which was sufficient to create the impression of a scene (or a real-world space) in their mind’s eye. This step enabled the structure of these core scenes (in terms of the proportion of SD, SA and background items contained within them) to be identified. Next, they were asked to remove the core items (one at a time) until just one item was left. Participants were informed that this would cause the scene to disintegrate but that they should attempt to remove the items in such a way that some impression of the scene was preserved for as long as possible. Participants marked the order in which they removed each item and highlighted the last remaining item. The proportion of SD, SA and background (Experiment 2) items retained last by participants in each of the scenes was calculated. This enabled a comparison across the three item categories to be made. As in the scene construction process, trials where participants were unable to deconstruct a scene were excluded (trials excluded: Experiment 1, 0%; Experiment 2, 0.83%).

### 2.5 Data analysis

The data were analysed using paired *t*-tests. In the case of the ranked scene construction data, the Wilcoxon signed-rank test for repeated measures data was utilised. Alpha was set at 0.05, and all analyses were corrected for multiple comparisons using the Bonferroni correction.

## 3 Results

### 3.1 Experiment 1

#### 3.1.1 Scene construction

**Fig. 3:**
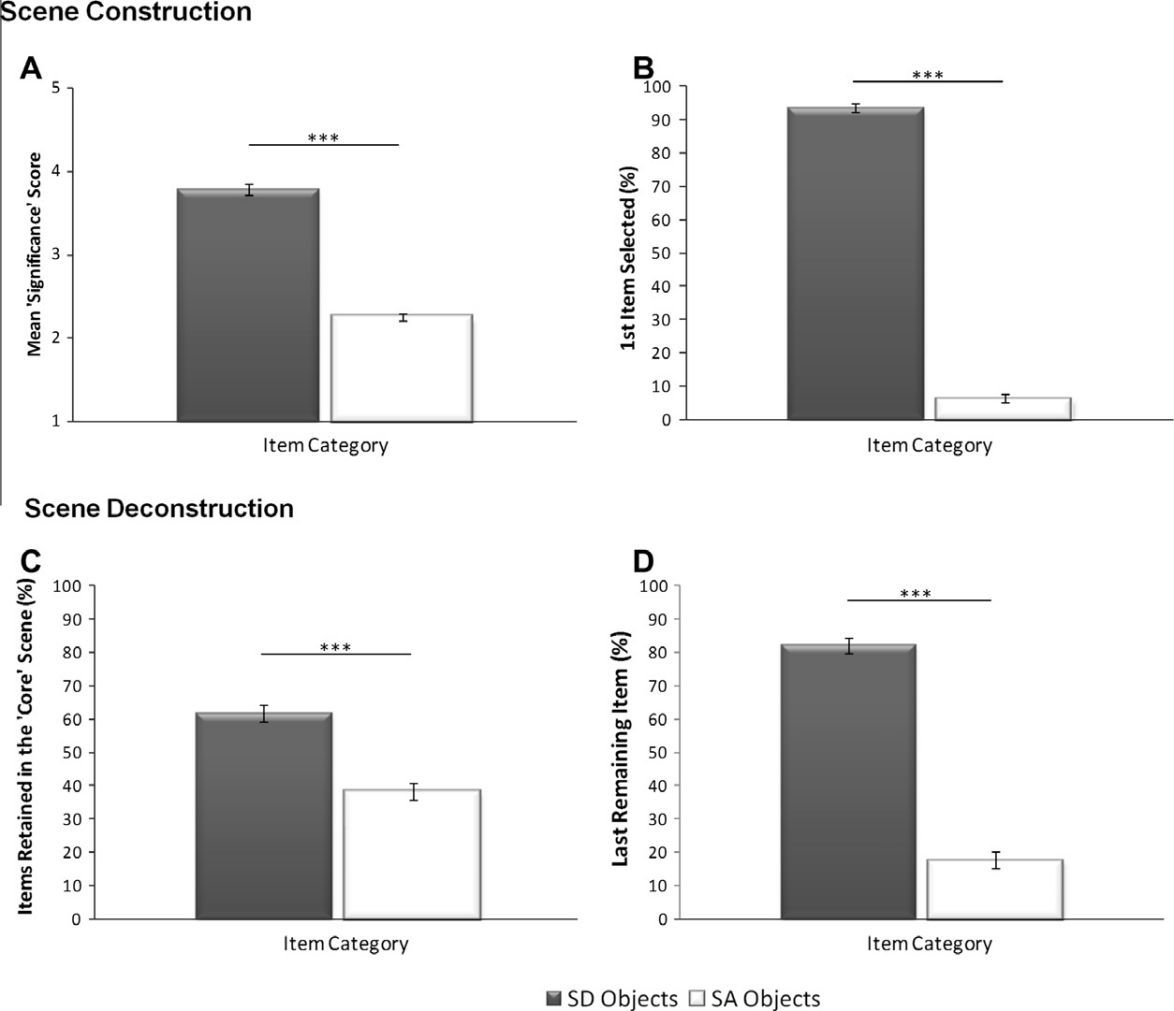
Results of Experiment 1. Scene construction: (A) Average ‘significance’ scores (±SE) for the SD and SA object categories. (B) Percentage of participants who selected an SD or an SA object first in the scene construction process. Scene deconstruction: (C) Percentage of SD and SA objects retained in ‘core scenes’. (D) Percentage of participants who selected an SD or an SA object as the last remaining item in the scene deconstruction process. ^***^p < 0.001.

SD objects scored higher (mean = 3.79, StD = 0.31) than SA objects (mean = 2.25, StD = 0.18; *z* = −3.92, *p* < 0.001), suggesting that SD objects were selected earlier than SA objects in the scene construction process (Fig. 3A). As the proportion of SD and SA objects in each scene was balanced across the 20 scenes, this preference for SD objects could not be attributed to an over-representation of SD objects. In addition to the mean significance score, we also explored the item selected first (and therefore considered to be the most critical) in the scene construction process. Overall, 93.75% of the items selected first were SD objects (StD 5.59%). Thus, SD objects were significantly more likely to be selected first in the scene construction process relative to SA objects (*t*(19) = 35, *p* < 0.001; Fig. 3B).

#### 3.1.2 Scene deconstruction

An average of 1.64 (out of a total of 5; StD 0.20) items were discarded initially, suggesting that 3.36 items were required to maintain a scene-like representation. Next, and in order to explore the structure of these ‘core’ scenes, the proportion of SD and SA objects retained relative to the total number of objects in the core scenes was calculated. Again a clear pattern emerged, whereby the core scenes contained a significantly greater proportion of SD objects (mean 61.72%, StD 10.68%) than SA objects (mean 38.28%, StD 10.68%; *t*(19) = 4.91, *p* < 0.001; Fig. 3C). Finally, we assessed the proportion of SD objects retained as the last item in the scene deconstruction process relative to the proportion of SA objects retained. This analysis revealed that once the ‘core’ scene structure was peeled away item-by-item, SD objects (mean 82.25%, StD 10.45%) were significantly more likely to be retained as the final item relative to the SA objects (mean 17.75%, StD 10.45%; *t*(19) = 13.81, *p* < 0.001). This shows that not only do SD objects maintain their dominance over SA objects in the core scenes, but that they were also consistently selected as the item most critical in preserving the scene, or an impression of real-world space, when participants were forced to discard all other objects from the scene (see Fig. 3D).

#### 3.1.3 Additional analyses

SD objects are typically larger than SA objects. We therefore wondered whether size alone was driving the effects we observed. We examined how participants dealt with the larger, free-standing SA items that we had included in some scenes (i.e. ‘a modern chrome floor light’; ‘a soft corduroy bean bag’). Notably, despite their larger size, these items were not treated differently from the SA group as a whole in the scene construction process (i.e. mean scene construction significance score: floor light = 2.25; bean bag = 2.45; all SA objects = 2.25) and were readily discarded by participants when selecting items for their core scenes (percentage of participants who discarded each item: floor light = 55%; bean bag = 65%). SD objects therefore seem especially important to scenes, and this is not entirely explicable in terms of their size.

In another analysis, we used the ratings acquired in our original study (Mullally & Maguire, 2011) to identify the largest SD object and the smallest SA object in every scene. We then calculated the proportion of these ‘largest SD objects’ selected first in the scene construction process, which was 57.75% (i.e. 57.75% of the objects selected first were the largest SD objects), and compared this to the total proportion of all SD objects (regardless of size) selected first in the scene construction process (93.75%). Thus, 36% of the SD objects selected first in the scene construction process were not the largest SD object available to participants, suggesting that although clearly important, size alone is not the sole determinant of participants’ selections. When we performed the same analysis for the SA objects – we identified the proportion of the ‘smallest SA objects’ selected last in the scene construction process (33%) relative to the total number of SA objects selected last in the scene construction process (76%) – we found that over half the SA items selected last in the scene construction process (and therefore considered the least important) were not the smallest SA objects. Thus, participants’ decisions were not being driven by object size alone.

### 3.2 Experiment 2

#### 3.2.1 Scene construction

**Fig. 4:**
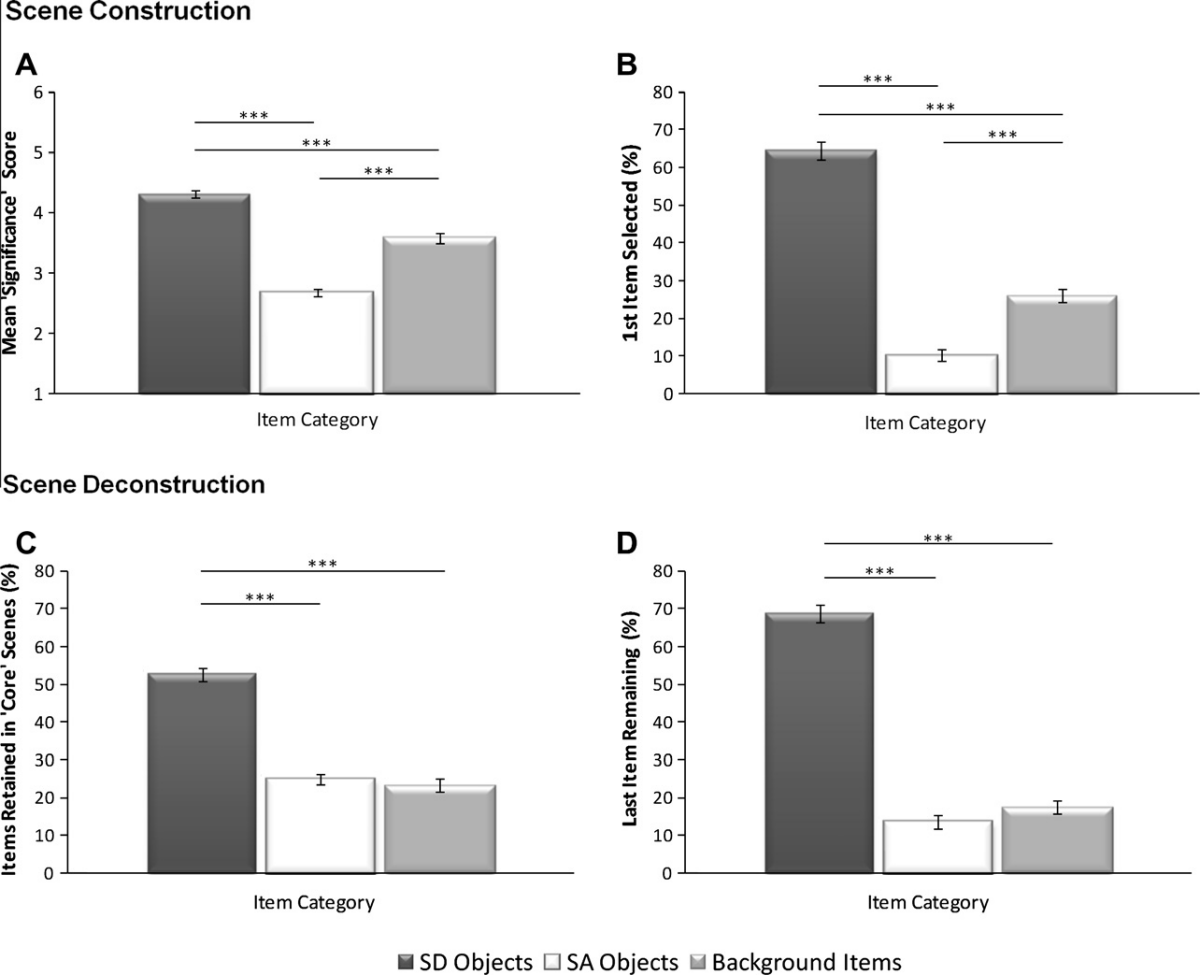
Results of Experiment 2. Scene construction: (A) Average ‘significance’ scores (±SE) for each of the three item categories. (B) Percentage of participants who selected an SD, SA or background item first in the scene construction process. Scene deconstruction: (C) Percentage of SD, SA and background items retained in ‘core scenes’. (D) Percentage of participants who selected an SD, SA, or background item as the last remaining item in the scene deconstruction process. ^***^p < 0.001.

Just as in Experiment 1, SD objects had a greater mean significance (mean 4.31, StD 0.38) than SA objects (mean 2.68, StD 0.35; *z* = −4.72, *p* < 0.001). Moreover, the SD objects’ score was also greater than the mean significance score attained by the background items (mean 3.58, StD 0.40; *z* = −4.31, *p* < 0.001), despite the fact that background items scored significantly higher than SA objects (*z* = −4.6, *p* < 0.001). Therefore, SD objects were selected earlier in the scene construction process than either SA objects or background items (Fig. 4A), and critically this preference for SD objects was preserved when equal numbers of items categories were presented to participants (i.e. when the ten ‘Type 3’ scenes, which contained 2 SD objects, 2 SA objects, and 2 background items each, were analysed separately; SD > SA: *z* = −2.70, *p* < 0.05; SD > backgrounds: *z* = −2.55, *p* < 0.05; backgrounds > SA: *z* = 2.50, *p* < 0.05.

Similarly, SD objects (mean 64.42%, StD 12.50%) were significantly more likely to be selected first in the scene construction process, relative to both SA objects (mean 10.25%, StD 8.06%; *t*(29) = 17.05, *p* < 0.001) and background items (mean 26.00%, StD 9.53%; *t*(29) = 10.51, *p* < 0.01; Fig. 4B), whilst background items scored significantly higher than SA objects (*t*(29) = −6.18, *p* < 0.001). Again this pattern was evident when the ten ‘Type 3’ scenes were analysed separately SD > SA: *t*(9) = 8.78, *p* < 0.001; SD > backgrounds: *t*(9) = 5.15, *p* < 0.01; backgrounds > SA: *t*(9) = −4.75, *p* < 0.01). Therefore, SD objects appeared to robustly maintain their dominance in the scene construction process, even when background items were available to participants. Moreover, background items, although not as dominant as SD objects, appeared to play a more critical role in creating an impression of a real world space than SA objects.

#### 3.2.2 Scene deconstruction

Participants discarded initially an average of 2.4 items (out of a total of 6 items), leaving an average of 3.60 items (StD 0.27) in the core scenes, comparable with Experiment 1. Moreover, and as before, the core scenes selected by participants contained a significantly larger proportion of SD (mean 52.64%, StD 9.2%) relative to SA objects (mean 24.89%, StD 7.26%; *t*(29) = 10.70, *p* < 0.001). Moreover, the proportion of SD objects in the core scenes was greater than the proportion of background items [mean 23.26%, StD 8.94%; *t*(29) = 10.18, *p* < 0.001; Fig. 4C). There was no difference in the ratio of background items and SA objects (*t*(29) = 0.66, *p* = 0.51). When the ten ‘Type 3’ scenes were analysed alone, a similar pattern was evident (SD > SA: *t*(9) = 10.72, *p* < 0.001; SD > backgrounds: *t*(9) = 5.10, *p* < 0.01] with the only exception being that here there was a significantly greater proportion of background items relative to SA objects [backgrounds > SA: *t*(9) = −4.51, *p* < 0.01]. Therefore, the core scenes selected by participants contained more SD objects than either SA objects or backgrounds items, and this was true irrespective of the original ratio of items categories within the given scenes. Moreover, background items, when presented in equal proportions to SD and SA objects (i.e. in ‘Type 3’ scenes), were more frequently represented in the core scenes than SA objects.

Finally, when we examined the proportion of SD, SA, and background items retained as the final item, a clear preference for the retention of SD objects (mean 68.8%, StD 12.61%) over both SA objects (mean 13.52%, StD 9.92%; *t*(29) = 14.73, *p* < 0.001) and background items (mean 17.35%, StD 9.7%; *t*(29) = 13.91, *p* < 0.001; Fig. 4D) was evident. No differential preference for background items over SA objects was observed (*t*(29) = −1.41, *p* < 0.168). An identical pattern of results was attained when the ‘Type 3’ scenes were analysed separately (SD > SA: *t*(9) = 10.0, *p* < 0.001; SD > backgrounds: *t*(9) = 7.0, *p* < 0.001; backgrounds > SA: *t*(9) = −1.65, *p* = 0.13]. Thus, participants showed a clear preference for SD objects over both background and SA items when they were forced to discard all other items from the scene.

#### 3.2.3 Background items – walls or floors

An additional analysis was possible in Experiment 2. In 20 scenes (10 ‘Type 1’ and 10 ‘Type 2’), half contained a single floor item (e.g. ‘a pale patterned carpet’), and half contained a single wall item (e.g. ‘a light yellow wall’). By collapsing ‘Type 1’ and ‘Type 2’ scenes together, and using the floor/wall distinction as the dependent variable, the significance of ‘wall’ and ‘floor’ background items could be directly compared across each of the four measures discussed above. Interestingly, we observed no difference between these two types of background items either when participants were constructing (mean significance scores: *t*(18) = 0.1, *p* = 0.99; first item selected: *t*(18) = −1.48, *p* = 0.157) or deconstructing (core scenes: *t*(18) = 1.18, *p* = 0.25; last item remaining: *t*(18) = −0.30, *p* = 0.76) their scenes. This suggests floors and walls were treated similarly in the construction and maintenance of scene representations, and that neither background type alone was capable of superseding the importance of SD objects in these processes.

## 4 Discussion

When viewed or imagined in isolation, SD objects evoke a strong sense of three-dimensional local space, and produce significantly greater activity in bilateral posterior parahippocampal cortex than SA objects (Mullally & Maguire, 2011). This region, often referred to as the parahippocampal place area (Epstein & Kanwisher, 1998), has long been associated with the processing of scene stimuli. The comparable neural response within PHC to both full scenes and single SD objects, led us to hypothesise that SD objects might play a more critical role in the construction and maintenance of scene representations than SA objects. The results of both Experiments 1 and 2 strongly supported this hypothesis, with participants choosing SD over SA objects as the first and most critical item in their constructed scenes and, more generally, selecting SD objects earlier than SA objects across the scene construction process. Similarly, when participants had to discard the objects they considered to be epiphenomenal to the maintenance of a coherent scene representation, leaving just the essential ‘core scene’, we consistently found that participants retained significantly more SD objects than SA objects. This strongly suggests that SD objects were considered to be more essential in the maintenance of scene-like representations than SA objects. Finally, the significance of a single SD object over a single SA objects within a scene context was reinforced by examining the final item which participants chose to retain once they had discarded all other objects from their scene. Here we found that the last remaining object across all scenes was most likely to be an SD object. Thus, even though participants were unaware of the SD/SA distinction, SD objects were considered by participants in both Experiments 1 and 2 to be more important in the initial construction and maintenance of a real-world scene than SA objects or background items such as walls and floors.

That SD objects could be important for the construction and maintenance of scene representations was an idea we deduced based on the results of Mullally and Maguire’s (2011) study. They found that the SD dimension of an object was best characterised in terms of two object properties, the object’s size and the permanence of its location within the environment. Therefore large objects, which rarely move, scored high on the SD dimension, whilst smaller, less permanent, objects tended to be rated as SA. While we found that size alone was not solely responsible for our findings, it is nevertheless understandable why SD objects could be fundamental constituents for scenes. SD objects provide scene elements which remain constant across consecutive saccades and different egocentric viewpoints. SD objects were clearly invoked as the key elements with which to construct scenes by participants in both Experiments 1 and 2.

Perhaps surprisingly, the introduction of background items (e.g. walls and floors) into the scenes in Experiment 2 did not detract from the superiority of the SD objects in the construction and maintenance processes. The opposite pattern could potentially have been expected for a number of reasons. First, the existence of cells such as “boundary vector cells” (Burgess, Jackson, Hartley, & O’Keefe, 2000; Hartley, Burgess, Lever, Cacucci, & O’Keefe, 2000; O’Keefe and Burgess, 1996) in the subiculum (Lever, Burton, Jeewajee, O’Keefe, & Burgess, 2009) and border cells in entorhinal cortex (Solstad, Boccara, Kropff, Moser, & Moser, 2008), which are hypothesised to index the animal’s position in relation to boundary items such as walls, thereby acting as the environmental input to hippocampal place cells, may suggest that background items such as walls should have of particular relevance for scenes. Moreover, it has previously been documented that the PHC responds equally to photographs of rooms containing furniture and the unfurnished versions of the same rooms (i.e. empty rooms with just bare walls) (Epstein et al., 1999). Given this, it could be argued that the most efficient strategy available to participants when constructing their scenes (particularly in the ‘Type 3’ scenes, where each scene contained both a wall and a floor item), would have been to firstly imagine the floor and wall items to create a space or ‘empty room’, which could later be populated with the SD and SA objects. However, previous fMRI data did not find evidence of a strong signal within PHC when participants imagined background items (Mullally & Maguire, 2011). This contrasted with the robust and extensive activations observed for SD objects in this same region (Mullally & Maguire, 2011). We therefore hypothesised that SD objects would be more relevant and considered more important to both scene construction and scene maintenance than background items.

It is possible, however, that when participants visualised an SD object, they implicitly imagined a floor beneath it, thus negating the requirement to add an explicit floor item. SD objects appear to exert their influence by anchoring themselves within their surrounding space and therefore inherently imply a surface below them (e.g. when you imagine a grand piano you imagine this anchored to a floor beneath it). Moreover, people often report that they feel like they could walk around an SD object, and therefore have a clear representation of the local space surrounding it. This experience is absent when one imagines a free-standing SA object such as a bean-bag or a tall floor light, as here participants report feeling that these objects (when imagined in isolation) are floating in space. Thus the key difference between an SD object and the floor elements presented in Experiment 2 is that an SD object only defines the local space around it, while a floor item should evoke a representation of the whole floor and thus a much larger space. If when creating a scene the floor is critical, then imagining a floor item should supersede imagining an SD object in our scene construction paradigm. Interestingly, this is not what we observed, and our results instead suggest that SD objects do something within a scene that floor items do not.

More specifically, we found that across each of our four measures in Experiment 2 (i.e. the scene construction mean significance score, the first item selected, the core scene composition, and the last item retained), participants rated SD objects as more relevant and considered them to be more important to both scene construction and scene maintenance than background items and this pattern was replicated when only the ‘Type 3’ scenes were interrogated (where the proportion of SD, SA, and background items was equal). That is, participants consistently selected background items later during scene construction and discarded them earlier during scene maintenance/deconstruction, than SD objects. We propose that the presence of SD objects, which are typically larger and permanently anchored within their environment, enabled participants to bypass the necessity to include boundaries such as walls or explicit floors within their scene constructs. This is because these SD items are theoretically capable of acting as the environmental input to hippocampal place cells (Bird, Capponi, King, Doeller, & Burgess, 2010). In addition, and when imagined in multiples, SD objects should automatically construct a sufficiently well-defined large-scale space, which would not be significantly enhanced by the addition of a boundary structure (e.g. a wall). Thus, in the presence of SD objects, boundary features such as walls and floors may not be necessary in attaining the impression of a real-world cohesive scene. Moreover, SD objects also have the advantage of offering, not only the impression of real-world three-dimension space, or of providing anchor points within that space, but they also add semantic details to the scenes. In this way, SD objects offer a unique combination of key scene attributes that may only be available singly from background items or SA objects.

We also wondered whether participants constructed (and deconstructed) scenes in a purely semantic sense without any regard to spatial considerations. Given the detailed instructions provided and feedback from participants post-testing, we feel that this was unlikely. Nevertheless, we also examined those of our stimuli that were acontextual, i.e. that did not give rise to a clear context such as a sitting room/bedroom/kitchen. Interestingly in these acontextual scenes participants’ reliance on SD objects early in the scene construction process was still evident. For example, the following scene was presented in Experiment 1 and contained three SD objects (a canvas director’s chair, a red snooker table, and a white free-standing bookcase) and two SA objects (a red photograph album and a large black torch). Here the first item selected by 60% of participants was the largest SD object (the red snooker table), which is in keeping with overall scene construction trends. Moreover, the mean significance scores of the three SD objects (a canvas director’s chair = 3.25; a red snooker table = 4.1; a white free-standing bookcase = 3.75; overall mean = 3.7) were comparable with the overall mean significance score for the SD objects in this experiment (3.79). In addition, these scores were clearly greater than those of the two SA objects (a red photograph album = 2.4; a large black torch = 1.5; overall mean = 1.95) and it is hard to see how the semantic value of the canvas director’s chair is greater than the semantic value of the photo album or the torch in this specific scene. Yet, the participants treated them differently, suggesting that semantic value alone cannot explain the clear difference in how participants weighted the importance of SD and SA objects when constructing scenes. Future experiments may illuminate spatial and semantic aspects of scenes further.

It is, however, worth noting that SA objects are not irrelevant to scenes. In fact 86.5% of the core scenes selected in Experiment 1, and 64.5% of the core scenes in Experiment 2, contained at least 1 SA object. Therefore, the core scenes, although dominated by SD objects, were never composed of SD objects alone. However, the proportion of SA relative to SD objects was always significantly less. Perhaps SA objects offered participants the opportunity to add finer detail to their scenes that may have helped to make a specific scene distinctive and more memorable. Nevertheless, whatever their function, SA objects did not aid scene construction or maintenance to the same degree as SD objects, or even background items. It would be interesting in future studies to investigate whether it is possible to construct a scene using SA objects only. If so, we hypothesise that such scenes would be phenomenologically different from a scene that contains both SD and SA objects, or SA and background items.

Finally, we observed an interesting parallel in the core scenes across Experiments 1 and 2. Despite starting with a different number of individual scenes items (5 in Experiment 1, and 6 in Experiment 2), participants in both experiments retained a similar number of items in their ‘core scenes’ (3.36 items in Experiment 1 and 3.6 items in Experiment 2). Participants therefore considered that representations containing between three and four items were sufficient for them to maintain a vivid and coherent scene in the mind’s eye. Similar behavioural data were reported by Summerfield et al. (2010) using a scene construction task (that did not consider SD/SA), leading those authors to conclude that the imagination of three combined scene elements was sufficient to attain a representation of a coherent, vivid scene. Moreover, using fMRI Summerfield et al. (2010) reported a distinctive biphasic pattern of activity during the scene construction process, whereby regions such as PHC, hippocampus, and retrosplenial cortex activated when participants imagined the first scene element, and then again when they combined the third scene element with the previous two items. Interestingly, this activity steadily declined with the addition of additional items into the scenes. Therefore, the addition of the third item to the scene represented a distinct time point that specifically engaged a core scene network of brain regions, most likely because this is the point when the true scene representation was formed by participants. This neuroimagining evidence, coupled with our participants’ willingness to discard the sixth, fifth and often fourth items from their scenes, further reinforces the idea that a scene need only contain between three to four scene elements for it to feel like a coherent representation of a real-world scene. Here, we were able to pinpoint the specific type of scene element necessary to attain this impression. We conclude that scenes, when stripped to their most basic structure, are dominated by items which provide participants with a sense of three-dimensional space, and it is this property which appears to be an essential building block of scenes.
